# Thon rings from amorphous ice and implications of beam-induced Brownian motion in single particle electron cryo-microscopy

**DOI:** 10.1016/j.ultramic.2015.05.017

**Published:** 2015-11

**Authors:** G. McMullan, K.R. Vinothkumar, R. Henderson

**Affiliations:** MRC Laboratory of Molecular Biology, Francis Crick Avenue, Cambridge CB2 0QH, UK

**Keywords:** Amorphous ice, Radiation damage, Thon rings, Markov Process, Noise whitening

## Abstract

We have recorded dose-fractionated electron cryo-microscope images of thin films of pure flash-frozen amorphous ice and pre-irradiated amorphous carbon on a Falcon II direct electron detector using 300 keV electrons. We observe Thon rings [1] in both the power spectrum of the summed frames and the sum of power spectra from the individual frames. The Thon rings from amorphous carbon images are always more visible in the power spectrum of the summed frames whereas those of amorphous ice are more visible in the sum of power spectra from the individual frames. This difference indicates that while pre-irradiated carbon behaves like a solid during the exposure, amorphous ice behaves like a fluid with the individual water molecules undergoing beam-induced motion. Using the measured variation in the power spectra amplitude with number of electrons per image we deduce that water molecules are randomly displaced by a mean squared distance of ∼1.1 Å^2^ for every incident 300 keV e^−^/Å^2^. The induced motion leads to an optimal exposure with 300 keV electrons of 4.0 e^−^/Å^2^ per image with which to observe Thon rings centred around the strong 3.7 Å scattering peak from amorphous ice. The beam-induced movement of the water molecules generates pseudo-Brownian motion of embedded macromolecules. The resulting blurring of single particle images contributes an additional term, on top of that from radiation damage, to the minimum achievable B-factor for macromolecular structure determination.

## Highlights

• Thon rings can be seen from amorphous ice.
• Radiation damage to amorphous ice randomly displaces water molecules.
• Each incident 300 keV e^−^/Å^2^ displaces water molecules on average by ∼1 Å.
• Macromolecules embedded in amorphous ice undergo beam induced Brownian motion.

## 1 Introduction

During the 1980s, Dubochet and his colleagues [2,3] developed a method for carrying out electron cryo-microscopy (cryoEM) of biological structures embedded in thin films of amorphous ice. Their early work involved careful comparison of the conditions needed to obtain thin films of amorphous rather than hexagonal or cubic crystalline ice [2]. The method they developed consisted of rapidly freezing a thin film of water, or buffer solution, containing the biological structure of interest by plunging it into liquid ethane at a temperature just above that of liquid nitrogen. Images of the resulting specimen recorded using a suitable microscope could then be analysed to determine the structure. The method has since grown immensely in its power and popularity as improvements in technology have transformed its capability [4].

There has long been a debate within the electron cryo-microscopy community as to whether Thon rings [1] can or should be observed in power spectra of images of pure plunge-frozen amorphous ice. Thon rings arise naturally in bright-field phase contrast images of any amorphous material and their origin is described in most standard texts [5,6]. The spacing and the shape of the rings depend on the electron–optical parameters that describe the image, principally the amount of defocus and astigmatism. Thon rings from amorphous carbon are routinely used to adjust astigmatism and set the defocus of a microscope. Amorphous ice has approximately one-half the density of amorphous carbon but as oxygen atoms scatter more strongly than carbon atoms [5] one might naively expect to see Thon rings of similar strength from amorphous ice and amorphous carbon.

In this paper we show that with the recently introduced CMOS direct electron detectors it is possible to see unambiguously Thon rings in images of high purity amorphous ice. The higher detective quantum efficiency, DQE, of the new detectors allows weaker signals to be seen and the ability to collect images continuously in a dose fractionated (or movie) mode allows optimal exposure conditions to be chosen and new modes of image processing to be used, long after the sample has been removed from the microscope.

## 2 Theory

### 2.1 Definitions

In this work the pixel value in an image at pixel (*r*,*s*) is denoted $f_{r,s}$, and scaled to units of e^−^/pixel. The discrete Fourier transform, f(u), at spatial frequency u is calculated using

(1) $$f\left(u\right)=\sum_{r,s}f_{\mathrm{rs}}\exp\left(-i2\pi u\cdot x_{\mathrm{rs}}\right)$$

and the power spectrum, S(u), is calculated using the convention.

(2) $$S(u)=\frac{1}{N_{\mathrm{tot}}}|f(u){|}^{2}=\frac{1}{N_{\mathrm{tot}}}{\left|\underset{r,s}{\sum }f_{\mathrm{rs}}\exp(-i2\pi x_{\mathrm{rs}}.u)\right|}^{2}.$$

where *N*_tot_ is the total number of pixels.

### 2.2 Noise whitened power spectra

The modulation transfer function, MTF, and detective quantum efficiency, DQE, of the Falcon II detector are given in [7]. The MTF of the Falcon II drops rapidly with increasing spatial frequency corresponding to the Falcon II having a broad point spread function, PSF. For the combination of pixel size and sensitive layer thickness used in the Falcon II the PSF is chiefly determined by charge carrier diffusion in the sensitive layer rather than the scattering of incident electrons [7]. As a result the DQE of the Falcon II does not fall with the MTF. The strong spatial frequency dependence of the MTF is however reflected in the power spectra from the Falcon II and can make it difficult to see weak signals. In this case it is advantageous to use the noise whitened power spectrum( NWPS), W(u), given by

(3) W(u)=S(u)/N(u)

in which N(u) is the normalised noise power spectrum [7]. For uniformly illuminated images, noise whitening removes the spatial frequency dependence of S(u) by boosting the signal at higher spatial frequencies. In particular it gives a flat background on which small signals can more easily be seen.

In [7] it was shown that for the Falcon II, N(u) could be estimated directly from the measured modulation transfer function, MTF. Using N(u) estimated in this way results in only a few percent variation in W(u) over the range from near zero to the Nyquist frequency in images with no sample present. In the present work it was found that the residual variation in W(u) with u could be further reduced by using a fit to the measured S(u) with a model for N(u) consisting of only a radially symmetric function and its low order aliased terms. Note that while N(u) is a two-dimensional function almost all of the non-circularly symmetric components arise simply from the contributions of aliased terms. Details of the fitting procedure are given in Section E of the Supplementary material.

### 2.3 Probability distribution for values in the noise whitened power spectra

For uniformly illuminated images the probability distribution for values of W(u) can be described using a single parameter. With no sample, the real and imaginary components of f(u) in Eq. (1) are independent Gaussian random variables [8]. As a result the squares of the real and imaginary components have *χ*^2^ distributions and their sum, S(u), an exponential distribution. Noise whitening sets the same average value, and hence exponential distribution for values of W(u), at all spatial frequencies, u. For any given spatial frequency, u, the probability that W(u) has value *y* is therefore

(4) $$\mathrm{Prob}\left[W\left(u\right)=y\right]=\exp\left(-y/\Gamma \right)/\Gamma$$

in which *Γ* is the mean value of W(u).

The DQE of the Falcon II detector is principally limited by the intrinsic variability of its response to individual electrons. If the average gain and the variance in the gain are $\overset{¯}{g}$ and *σ*_*g*_^2^, respectively, then the value of noise power spectrum in the limit of zero spatial frequency with *d* incident independent electrons per pixel is [9]

(5) $$S\left(0\right)={\bar{g}}^{2}d+\sigma_{g}^{2}d$$

and

(6) $$\mathrm{DQE}(0)={\overset{¯}{g}}^{2}/({\overset{¯}{g}}^{2}+\sigma_{g}^{2}).$$

Using Eqs. (3)– (6) gives

(7) $$W\left(0\right)=W\left(u\right)=d/\mathrm{DQE}\left(0\right)=\Gamma$$

since the average value of W(u) is the same for all u, by definition N(0)=1 and expressing the detector output in units of incident electrons sets the gain to unity. The probability distribution for values of W(u) depends solely on $\Gamma =d/\mathrm{DQE}\left(0\right)$ via Eq. (4) and has both mean and standard deviation of *Γ*. In particular, for the Falcon II DQE(0)∼0.5 [7] and so Γ~2d.

### 2.4 Power spectra of dose fractionated series

If a dose fractionated exposure consists of *M* frames with an average of *d* uncorrelated electrons per frame, from Eq. (7) the expected value of $W_{M,m}$ defined as the sum of the M/m noise whitened power spectra from the images obtained by summing *m* consecutive frames is

(8) $$W_{M,m}=\overset{M/m}{\underset{i}{\sum }}(\mathrm{md}/\mathrm{DQE})=\frac{D_{\mathrm{tot}}}{\mathrm{DQE}}.$$

in which $D_{\mathrm{tot}}=\mathrm{Md}$ is the total number of electrons in the exposure. Similarly the expected noise, $N_{M,m}$, in $W_{M,m}$ is

(9) $$N_{M,m}=\sqrt{\overset{M/m}{\underset{i}{\sum }}{\left(\mathrm{md}/\mathrm{DQE}\right)}^{2}}=\frac{D_{\mathrm{tot}}}{\mathrm{DQE}}\sqrt{\frac{m}{M}}.$$

While the value of $W_{M,m}$ is independent of *m*, the noise in sum, $N_{M,m}$, grows as the square root of *m*. In practice residual correlation in pixel values between frames, such as from an offset drift, will result in an *m* dependence in $W_{M,n}$. The actual Falcon II detector used here had a small correlated shift in the row-reset between successive frames that results in a 1% drop in $W_{M,m}(u)$ between *m*=1 and *m*=2. However the largest systematic correlation comes from the applied per pixel gain correction of the images which results in an increase in $W_{M,m}(u)$ with increasing *m* but with careful gain calibration keeps the effect to less than 5% in going from *m*=1 to *m*=120.

### 2.5 Effect of random motion of water molecules on power spectra

At any given instant there is typically only a single high energy electron interacting with the sample at the beam intensity used in cryoEM. An incident electron passing through a thin layer of amorphous ice will see a particular conformation of atoms but the radiation damage resulting from the interaction of the high energy electron with the amorphous ice will perturb the conformation of atoms in the ice [10]. Subsequent high energy electrons incident on the ice will see, and perturb, the atomic configurations resulting in a Markov-like process for the evolution of the atomic configuration. In reality possible atomic configurations in amorphous ice are strongly constrained by the ice rules of Bernal and Fowler [11]. The simplest approximation for the transition probability of atom positions in going from one conformation to the next is to use a Gaussian distribution depending solely on the number of high energy electrons incident per unit area between the configurations . In particular if after *n* electrons per unit area the position of the *i*th water molecule is $x_{i}(n)$, after a further *d* electrons per unit area the probability distribution for its position has a three dimensional Gaussian distribution

(10) $$P(x_{i}(n+d):x_{i}(n))=\frac{1}{{\left(2\pi d\sigma_{0}^{2}\right)}^{3/2}}\exp\{-\frac{|x_{i}(n+d)-x_{i}(n){|}^{2}}{2d\sigma_{0}^{2}}\}$$

in which $\sigma_{0}^{2}$ is the mean squared displacement in a given direction in response to a single incident electron per unit area.

Assuming an image consists of *M* frames and has a total exposure of *D* electrons per unit area, the power spectrum, S(u), at spatial frequency u in terms of the Fourier components, $f_{i}(u)$, of the *i*th frame is

(11) $$S\left(u\right)\propto \langle |\sum_{i=1}^{M}f_{i}\left(u\right){|}^{2}\rangle =\sum_{i,j=1}^{M}\langle f_{i}^{\;⁎}\left(u\right)f_{j}\left(u\right)\rangle$$

where 〈…〉 denotes an ensemble average. Since the average number of incident electrons per unit area in each frame is *d*=*D*/*M*, using Eq. (10) the |i−j|d incident electrons between *i*th and *j*th frames will randomly displace the water molecules so that

(12) $$\langle f_{i}^{⁎}(u)f_{j}(u)\rangle \propto d^{2}F_{0}{\left(u\right)}^{2}\exp(-\alpha_{u}|i-j|d)$$

in which $\alpha_{u}=2\pi^{2}\sigma_{0}^{2}u^{2}$ and *σ*^2^_0_ is the induced mean squared motion of a water molecules per incident electron though a unit area while $F_{0}{\left(u\right)}^{2}$ is defined as

(13) $$F_{0}{\left(u\right)}^{2}\equiv \frac{1}{{\mathrm{Md}}^{2}}\overset{M}{\underset{i=1}{\sum }}\langle |f_{i}(u){|}^{2}\rangle .$$

Substituting Eq. (12) into Eq. (11) and summing over *i* and *j* gives

(14) $$S(u)=d^{2}F_{0}{\left(u\right)}^{2}\times (\frac{2e^{-\alpha_{u}d}}{{\left(1-e^{-\alpha_{u}d}\right)}^{2}}\{(1-e^{-\alpha_{u}d})M+e^{-\alpha_{u}\mathrm{Md}}-1\}+M).$$

In cases where there is very little induced movement $\alpha_{u}\to 0$ so that $S(u)\to M^{2}d^{2}F_{0}{\left(u\right)}^{2}$ and since *d*=*D*/*M* we have $S(u)\propto D^{2}$. On the other hand if there is no correlation between frames then $\alpha_{u}\to \infty$ and $S(u)\to {\mathrm{Md}}^{2}F_{0}{\left(u\right)}^{2}$.

For an exposure with a total of *D* electrons but where the molecules are continuously moving we can let M→∞ subject to *Md=D*. The summation in Eq. (11) goes over to a double integral. Carrying the integrals, or setting *Md*=*D* and letting *d*→0 in Eq. (14) , gives

(15) $$S(u)=2F_{0}{\left(u\right)}^{2}[\alpha_{u}D+\exp(-\alpha_{u}D)-1]/\alpha_{u}^{2}$$

which in the limits of small and large *α*_*u*_ goes to $D^{2}F_{0}{\left(u\right)}^{2}$ and $2{\mathrm{DF}}_{0}{\left(u\right)}^{2}/\alpha_{u}$, respectively.

### 2.6 Estimation of beam induced movement from dose fractionated images

The amount of movement resulting from an incident electron can be estimated from the variation in Thon ring signal as a function of the number of incident electrons in an image. When the induced motion is smaller than a given spatial frequency the Thon ring modulation at that spatial frequency is expected to grow quadratically with the number of incident electrons in an exposure but as the induced motion becomes comparable with the spatial frequency the amplitude should grow more slowly. From Eq. (15) the power spectrum, *S*(*u*), at a spatial frequency *u*, is expected to vary with total number of electrons per unit area, *D*, as

(16) $$S(u)\propto 2[\alpha_{u}D+\exp(-\alpha_{u}D)-1]/\alpha_{u}^{2},$$

in which $\alpha_{u}=2\pi^{2}\sigma_{0}^{2}u^{2}$, and *σ*_0_^2^ is the mean-squared movement in a given direction per incident electron passing through a unit area. The actual Thon ring modulation does not appear explicitly in this equation but the Thon ring modulation enables the signal from the ice to be identified over the background noise in the measured power spectra. The measured spatial frequency dependence of *S*(*u*) will also include that of the detector but for the Falcon II detector this can be removed by using the noise whitened power spectra, *W*(*u*) as defined in Eq. (3).

From a single dose fractionated exposure it is possible to estimate *σ*_0_^2^ through the dose dependence present in Eq. (16). If an the exposure consists of *M* frames each with an average of *d* elections per unit area, the result of summing the original frames in blocks of *m* is to produce M/m frames with on average *md* electrons per unit area. Denoting the sum of the resulting M/m noise whitened power spectra by $W_{M,m}(u)$ and using Eq. (16) gives

(17) $$W_{M,m}(u)\propto \frac{2D_{\mathrm{tot}}}{\alpha_{u}}(\alpha_{u}\mathrm{md}+\exp(-\alpha_{u}\mathrm{md})-1)/\alpha_{u}\mathrm{md}$$

in which $D_{\mathrm{tot}}=\mathrm{Md}$. The behaviour of Eq. (17) with *m* depends only on $z=\alpha_{u}\mathrm{md}$ and is described by the functional form

(18) $$g(z)=(z+e^{-z}-1)/z.$$

This is a monotonically increasing function that initially varies linearly with *z* but plateaus towards 1 for large *z*. By fitting the non-linear behaviour of $W_{M,m}(u)$ as a function of *m* it is possible to estimate *α*_*u*_, and hence obtain *σ*_0_^2^.

## 3 Experimental

Quantifoil R1.2/1.3 Cu/Rh grids were used to make a thin film sample of double distilled water (18MΩcm conductivity). The grids were glow-discharged in air for 30–40 s and 3 μl of water applied in an environmental plunge-freeze apparatus [12]. Grids were blotted for 4–6 s and rapidly frozen in liquid ethane. The grids were transferred to Krios cartridges and imaged using a Falcon II direct electron detector on a FEI Titan Krios operated at 300 keV. A nominal magnification of 75,000× corresponding a calibrated value of 134,600× was used which results in a 1.04 Å sampling with the 14 μm pixels of the Falcon II detector. Imaging was performed using nano-probe mode with parallel illumination using a 70 μm C2 aperture and no objective aperture. A beam centred on and slightly larger than the Quantifoil hole was used. Astigmatism and beam tilt correction were performed at the imaging magnification. Images were recorded at 18 frames/s for either 8 or 17 s with either 2.33 e^−^/frame, or 0.85 e^−^/frame, respectively. The electron exposure per frame was determined from the screen current and average signal from the Falcon detector, with these being calibrated against a picoammeter as described previously [7].

The amorphous carbon control specimen was made in an Edwards 301 vacuum coating unit by evaporating carbon from an arc onto mica. The thickness of the carbon film was estimated to be 150 Å from the optical density of the carbon deposited on adjacent piece of filter paper. The carbon film was floated off the mica using a water bath and placed onto a Cu/Rh grid. The continuous carbon grid was examined using the Atlas component of the FEI EPU software. From the Atlas an area of the carbon film was selected in which the carbon film had no holes or wrinkles both in the grid square and surround grid squares. After pre-irradiation of the carbon, images were recorded for 1.5 s with the same magnification and number of incident electrons per frames as used for the ice sample. The stage was moved to adjacent areas in a raster (using 0.4 μm steps in *X* followed by 0.4 μm steps in *Y*). A total of 25 images from different but adjacent areas of carbon film were collected with the defocus for all images set as close as possible to a nominal 5000 Å by manually adjusting the focus at each position. Later evaluation using CTFFIND3 [13] showed that the defocus was actually 5990 Å, with 200 Å of astigmatism and ±90 Å defocus variation over the 25 images.

Images were recorded using the full 4k×4k output of the Falcon II detector with the individual frames of all the images being captured using an in-house data capture setup. In processing the exposures the individual frames were not aligned computationally.

## 4 Results

**Fig. 1:**
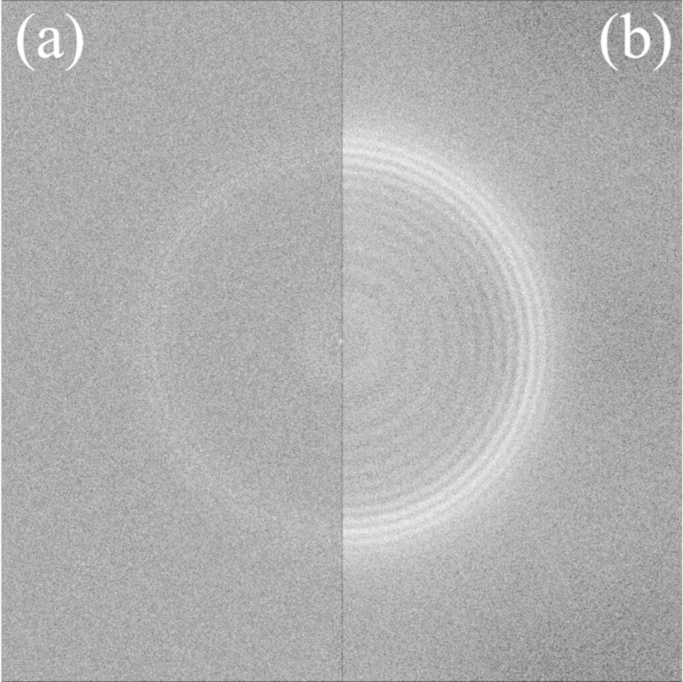
Thon rings from a dose fractionated exposure (image #230804) of amorphous ice obtained using either (a) the NWPS of the sum of the frames, or (b) the sum of the NWPS from each frame. The exposure consisted of 141 frames at a dose of 2.33 e^−^/pixel per frame recorded using 300 keV electrons, a defocus of 7070 Å and a pixel sampling of 1.04 Å. The edge of the transform is at 1/2.08 Å^−1^ and the strong Thon ring pattern in (b) is centred around 1/3.7 Å^−1^. In both (a) and (b) the images were first scaled so that the RMS noise level was 1 and then the lower and upper grey limits set at −0.7 and 1.3 about the mean values.

Fig. 1 shows two types of noise whitened power spectra (NWPS), W(u), [7] from a dose fractionated exposure of pure amorphous ice consisting of 141 frames with on average 2.33 e^−^/pixel per frame. The spectrum from the sum of all the 141 frames is shown in Fig. 1(a) and with such a relatively high dose (300 e^−^/Å^2^) it is possible to see faint Thon rings. Fig. 1(b) shows the sum of the 141 noise whitened power spectra of the individual frames. Exactly the same images were used but in the sum of the power spectra of the individual images the Thon rings that are barely visible in Fig. 1(a) are clearly visible and can be seen to extend out beyond 3.4 Å resolution. The $\sqrt{141}$ reduction in the noise from averaging the 141 individual spectra enables the strength of the Thon rings in individual frames to be seen clearly. The strength of this signal indicates that the Thon rings arise from the intrinsic bulk water and not from impurities absorbed from the adjacent carbon film or contamination occurring during the blotting and transfer steps.

The behaviour of the circularly averaged power spectra and circularly averaged noise whitened power spectra are compared in Section A of the Supplementary material. The flat, featureless backgrounds in Fig. 1(a) and (b) illustrate the success of the noise whitening procedure. The measured average value and noise in the noise whitened power spectra of Fig. 1 are within 5% of the values predicted by Eqs. (8) and (9) using *d*=2.33 and DQE(0)=0.5. The probability distribution based on all the values in the 141 noise whitened power spectra used in Fig. 1(b) is given in Section G of the Supplementary material. The distribution is well described by Eq. (4) with a measured value of Γ=4.91 that is within 5% of an estimated based on Eq. (7) again using *d*=2.33 and DQE(0)=0.5.

To illustrate the origins of the difference between Fig. 1(a) and (b) we carried out two control experiments using amorphous carbon film. The amorphous carbon film was first pre-irradiated as even atoms in films of carbon prepared by evaporation from a carbon arc *in vacuo* move when initially irradiated. The amount of movement decreases with exposure but ∼100 e^−^/Å^2^ is sufficient to effectively stabilise a film, i.e., the observed Thon ring pattern showed no drift and was stable. As 300 keV electrons can cause displacement damage [14], there was some residual movement but relative to the initial movement this was negligible.

**Fig. 2:**
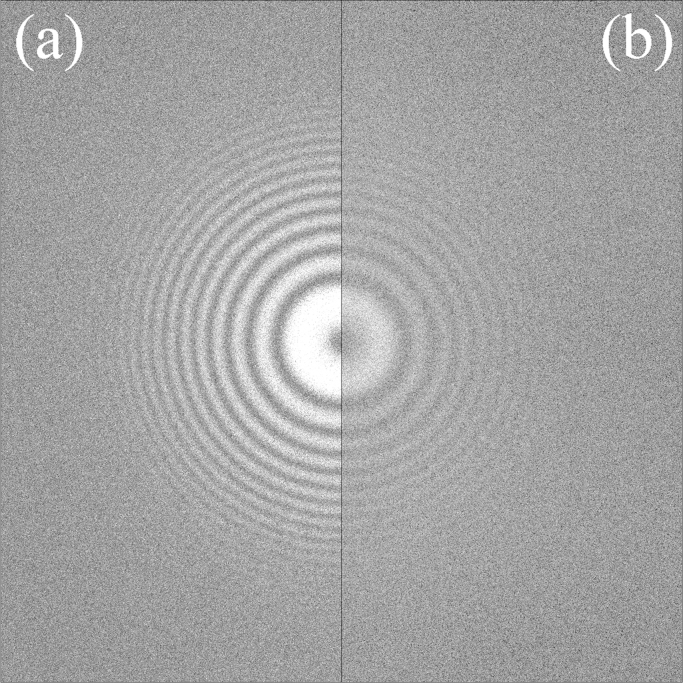
Comparison from a dose fractionated exposure (image #215327) of an area of pre-irradiated carbon of (a) the NWPS obtained from the sum of all the frames and (b) the sum of the NWPS of individual frames. The exposure consisted of 25 frames with an average of 2.33 e^−^/pixel per frame recorded using 300 keV electrons, at 5990 Å defocus and a 1.04 Å pixel sampling. The grey scales of the images were set as in Fig. 1.

In the first control experiment, the initial 25 frames from a 1.5 s exposure of an area of pre-irradiated carbon were used. The magnification and the number of incident electrons per frame were the same as in Fig. 1. The corresponding noise whitened power spectrum from the summed image and the sum of the individual power spectra are given in Fig. 2(a) and (b), respectively. In contrast to Fig. 1, the Thon rings are now stronger in the power spectrum of the sum of the frames with the relative strengths being essentially what is expected from 25 images of the same object.

**Fig. 3:**
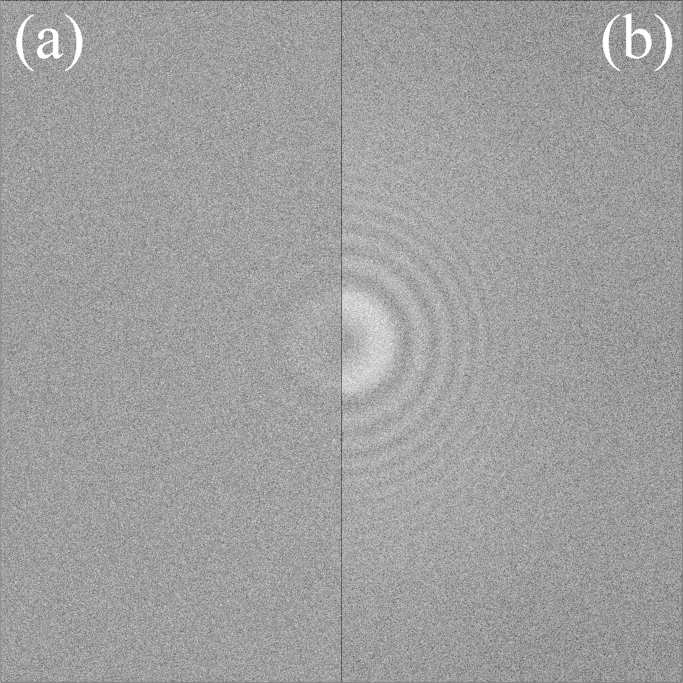
Comparison from a composite exposure of (a) the NWPS obtained from the sum of all the frames and (b) the sum of the NWPS of the individual frames. The composite image consisted of 25 single frames taken from different dose fractionated images of non-overlapping areas in a region of pre-irradiated amorphous carbon. The imaging conditions were the same as in Fig. 2 with the grey scales of the images set as in Fig. 1.

In the second control experiment, a series of 25 images like that in Fig. 2 were taken at adjacent but non-overlapping areas of the pre-irradiated carbon. As there was a slight variation in the height of the carbon film at the different locations the objective lens current was adjusted in order to keep the variation in the defocus of the images to within ±90 Å. A composite image of 25 frames was then generated by taking one frame from each of the 25 images. As in Figs. 1 and 2 the corresponding power spectra from the Fourier transform of the sum of the frames from the composite image is given in Fig. 3(a) while that of the sum of the power spectra of the individual frames in the composite image is shown in Fig. 3(b). Like Fig. 1, the Thon rings in Fig. 3 are more visible in the sum of the power spectra than in the power spectrum of the summed image. As expected Fig. 2(b) and Fig. 3(b) are almost identical since they both consist of the sum of 25 power spectra from images of areas with the same thickness of stabilised carbon taken with essentially the same defocus and number of electrons.

In Fig. 2, the carbon atom positions are effectively the same in all the frames while in Fig. 3 there no correlations between atom positions in the different frames. The similarity of Figs. 1 to 3 indicates that water molecules in amorphous ice are moving to uncorrelated positions during an exposure.

**Fig. 4:**
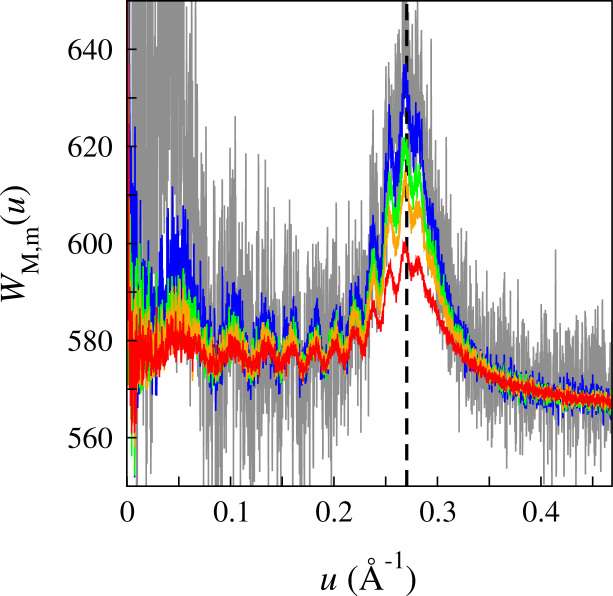
Circularly averaged power spectra, $W_{120,m}(u)$ as a function of spatial frequency, *u*, from 120 frames of image #230804 used in Fig. 1 showing the behaviour with *m*. Results for *m*=1 (red), *m*=2 (orange), *m*=3 (green), *m*=8 (blue), *m*=120 (grey) are shown. Exposure #230804 had 2.33 e^−^/pixel/frame and using Eq. (8) with a DQE of 0.5 for the Falcon II gives an expected average background Γ=560. The expected noise in the limit of zero spatial frequency from Eq. (9) is $51\sqrt{m}$ but this decreases with the square root of the spatial frequency as more values are included in the circular average. The vertical dashed line indicates the position of 1/3.7 Å data used in Fig. 5.

Fig. 4 shows the circularly averaged values of $W_{M,m}(u)$ at selected values of *m* obtained from the dose fractionated image series of amorphous ice in exposure #230804 used in Fig. 1. For simplicity in factorisation, only the first 120 of 141 frames were used. The average background value, the magnitude of the associated noise and their variation with *m* are in agreement with the predictions of Eqs. (8) and (9). There are some residual correlations within, and between, frames that lead to small systematic variations in $W_{M,m}(u)$ with *m*. These were removed by adding a small *m* dependent shift for each m>1 to ensure that at the Nyquist frequency (in this case 1/2.08 Å) $W_{M,m}(u)=W_{M,1}(u)$. The maximum shift required was for *m*=120 and corresponded to 5% of the background.

**Fig. 5:**
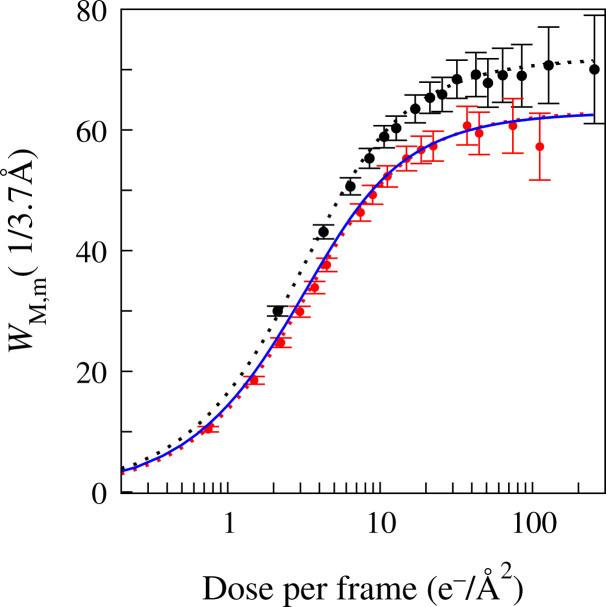
Measured contribution in the circularly averaged $W_{M,m}(u)$ from ice at $u=1/3.7\;\overset{˚}{A}$ as a function of the number of electrons per unit area in the summed frames. The results shown using black bullet (•) are from the first 120 frames of exposure #230804 with 2.33 e^−^/pixel/frame. The results shown using red bullet () are from the first 300 frames of exposure #171528 taken with the same magnification and defocus but over 15 s using 0.85 e^−^/pixel/frame on a different amorphous ice sample. The red and black dotted lines show fits to the corresponding experimental data using Eq. (17). The solid blue curve lies almost on top of the dotted red curve and is simply 0.87 times the dotted black curve. The error bars are based on circularly averaged estimates using Eq. (9).

Fig. 5 shows the circularly averaged values of $W_{M,m}(u)$ as a function of *m* at *u*=1/3.7 Å (indicated by the vertical dashed line in Fig. 4) from 120 frames of exposure #23804. Also shown in Fig. 5 are results from a dose fractionated image (#171524) consisting of 300 frames from a different amorphous ice sample using the same magnification as image #23804 but with an exposure rate of 0.85 e^−^/pixel per frame. The dotted lines in Fig. 5 are fits to the measured results using Eq. (17) and two adjustable parameters: *σ*_0_^2^ and a scale factor. The scale factor includes the electron optical terms and is proportional to the total number of electrons in the exposure, the scattering strength and thickness of the amorphous ice sample. Fits to successive dose fractioned images of a fixed area of amorphous ice give essentially the same value for *σ*_0_^2^ but the scale factor decreases as the sample is thinned by radiolysis. The fitted values of *σ*_0_^2^ shown in Fig. 5 for images #230804 and #171524 are 0.38 and 0.35 Å^2^/(e^−^/Å^2^), respectively. There are many sources of systematic error in this analysis and at best these values for *σ*_0_^2^ should be considered as estimates. Based on these estimates the radiation damage resulting from one incident 300 keV e^−^/Å^2^ is expected to induce a total mean squared motion of ∼1.1 Å^2^ in the water molecules of the sample.

For a dose fractionated exposure of *M* frames the Thon ring signal given by Eq. (18) (and illustrated in Fig. 5) initially increases linearly with *m* but plateaus at higher *m*. As the corresponding noise, given by Eq. (9), grows as $\sqrt{m}$ there is an optimal *m*, and hence dose per image, with which to observe Thon rings. Dividing Eq. (18) by $\sqrt{z}$ and setting the derivative with respect to *z* to zero gives a transcendental equation with zero at 2.149. For a given resolution, *u*, the optimal number of electrons per unit area, $d_{\mathrm{opt}}$, in a frame for observing Thon rings is

(19) $$d_{\mathrm{opt}}=2.149/2\pi^{2}\sigma_{0}^{2}u^{2}.$$

The peak is not very sharp but for a given dose the Thon rings around 1/3.7 Å from amorphous ice (assuming $\sigma_{0}^{2}=0.37\;{\overset{˚}{A}}^{2}/(e^{-}/{\overset{˚}{A}}^{2})$) will have the highest signal to noise ratio if each frame has, or successive frames are grouped into blocks with an average exposure of $∼4.0\;e^{-}/{\overset{˚}{A}}^{2}$.

**Fig. 6:**
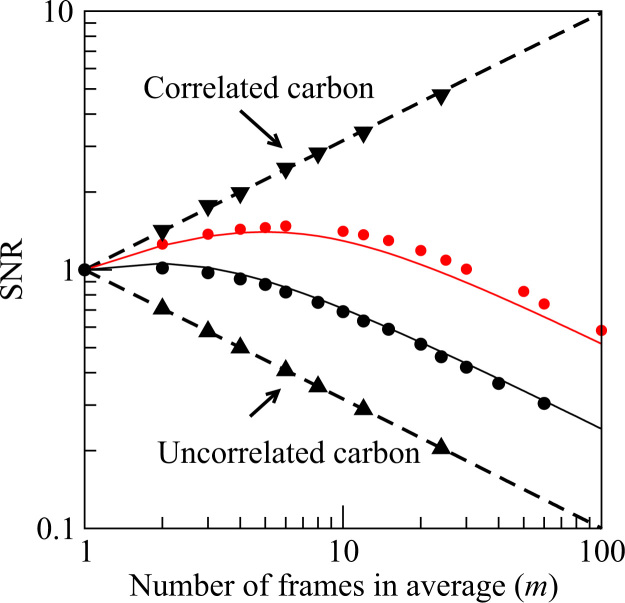
Relative signal-to-noise ratio of Thon rings in the sum of noise whitened power spectra, $W_{M,m}/W_{M,1}$, from a dose fractionated exposure of *M* frames as a function of the number of successive images in each power spectrum, *m*. Results at *u*=1/9.0 Å from 24 frames of the pre-irradiated carbon images used for Figs. 2 (▾) and 3 (▴) are shown. Results at *u*=1/3.7 Å from 120 frames of image #23804 (•) with 2.33 e^−^/pixel and 300 frames of image #171524 () with 0.85 e^−^/pixel of Fig. 5 are shown. The results using Eq. (9) for the noise and Eq. (18) for the signal with $\sigma_{0}^{2}=0.37\;{\overset{˚}{A}}^{4}/e^{-}$ are shown as solid lines.

Finally, while Fig. 5 gives the amplitude of the observed Thon ring modulation in $W_{M,m}(u)$ as *m* (and hence the exposure per image) is varied, it is also useful to look at how the corresponding visibility of the Thon ring modulation, i.e., the signal-to-noise ratio, varies. Fig. 6 shows a log–log plot of the variation in the signal-to-noise relative to the corresponding case with *m*=1 in the noise whitened power spectra as a function of *m*. Results are shown for both the amorphous ice exposures shown in Fig. 5 as well as the pre-irradiation carbon cases shown in Figs. 2 and 3. The results for amorphous ice fall between two extremes, given by a line varying as $\sqrt{m}$ for the case where the images are of the same object in each frame (such as in Fig. 2 for the images of the same area of pre-irradiated carbon), and in the other extreme of a line varying as $1/\sqrt{m}$ for the case where the images are of uncorrelated objects in the different frames (such as in Fig. 3 made up of images of different areas of amorphous carbon). The $\sqrt{m}$ and $1/\sqrt{m}$ limits indicated by the dotted lines in Fig. 6 simply follow from noting $W_{M,m}$ as defined in (17), varies as *m* in the limit where there is no movement between frames but goes to a constant when there is no correlation between frames while the noise given by Eq. (9) varies as $\sqrt{m}$. The signal-to-noise ratio curves for both exposure #23804 and #151524 initially vary as $\sqrt{m}$ but make the transition to $1/\sqrt{m}$ for large *m*. As expected from Eq. (19) in going from behaving as $\sqrt{m}$ to $1/\sqrt{m}$ the amorphous ice curves reach a peak at *m* corresponding to ∼4 e^−^/Å^2^. The solid lines in Fig. 6 show the predicted behaviour of $g(z)/\sqrt{z}$ in which *g*(*z*) is defined in Eq. (18) with $z=2\pi^{2}\sigma_{0}^{2}u^{2}\mathrm{md}$ at $u=1/3.7\;\overset{˚}{A}$ with $\sigma_{0}^{2}=0.37\;{\overset{˚}{A}}^{2}/(e^{-}/{\overset{˚}{A}}^{2})$ and *d*=2.33 e^−^/frame (black —) or *d*=0.85 e^−^/frame (red —).

## 5 Discussion

The recent introduction of higher performance electron detectors has allowed structures of biological macromolecules to be obtained by single particle cryoEM to higher resolution, with fewer particles and more easily than before [4]. However, these reconstructions still require averaging substantially more particle images than predicted to be necessary by theory [15]. Charging of the specimen and its surroundings as well as physical movement of the specimen during exposure to the first few e^−^/Å^2^ are two reasons for this discrepancy.

To observe well defined Thon rings from images of amorphous ice the sample must be neither too thick nor too thin. If the ice film is too thin, the amplitude of the Thon ring signal will simply be too small to be seen among the background noise. If the ice film is too thick the Thon signal will also become weaker, especially at high resolution. Part of this reduction comes from the variation in defocus as images of molecules at different heights have Thon ring zeroes at different spatial resolutions, and part is due to increased multiple elastic and inelastic scattering of the electron beam. If an ice film is too thick it is possible to use radiolysis to thin the film to the desired thickness by recording a succession of images.

The thickness of the ice used in Fig. 1 was not measured explicitly, however using the observation of every 170 e^−^/Å^2^ of 300 keV electron exposure reducing the thickness of an ice film by ∼100 Å [16], we estimate from the number of successive images required to completely burn through the ice film that the thickness was between 1000 and 1500 Å. The observed Thon ring modulation (measured as one half the peak to peak value divided by the average above the background) around 1/3.7 Å in the circular average of Fig. 1 is 9%. Thon ring modulation is attenuated with increasing spatial by partial coherence, beam divergence and variation in defocus through a sample. For a sample of thickness, *t*, the damping due solely to variation in defocus varies as sin(ξ)/ξ in which $\xi =\pi \lambda u^{2}t$ and *λ* the electron wavelength *λ* [17] (see Supplementary material Section D). For a sample of 1250 Å at a resolution of $u=1/3.7\;\overset{˚}{A}$ with 300 keV electrons ξ=5.65 and so sin(ξ)/ξ=−0.1. The magnitude of the predicted damping is similar to that observed but the negative sign coming from *ξ* being greater than the first zero of sin(ξ)/ξ would imply that there should be a node in the Thon ring modulation at $1/\sqrt{\lambda t}∼1/5\;\overset{˚}{A}$. The absence of any nodes in the Thon ring modulation in the circularly averaged power spectra shown in Fig. 4 suggests that the thickness of the ice is less than $1/{\mathrm{ut}}^{2}=700\;\overset{˚}{A}$. In Section I of the Supplementary material it is shown that a good fit to the observed Thon ring modulation can be obtained with *t*=620 Å. The discrepancy between this value and the thickness estimate based on the rate of radiolysis thinning given above can be explained in many ways. The estimate from radiolysis thinning may be an overestimate due to the constant replacement of water molecules lost by radiolysis with molecules migrating in from the surround ice. On the other hand the estimate from the Thon ring modulation signal will underestimate the actual ice thickness as the contribution from the surface layers will be reduced due to the dynamic disorder resulting from the escape of radiolysis byproducts. The sin(ξ)/ξ behaviour is also approximate as the sample thickness is changing and the signal profile through the sample is not uniform.

The high number of electrons used in Fig. 1 (304 e^−^/Å^2^) ensures that the Thon rings are clearly visible but is not necessary. With the correct conditions Thon rings can easily seen with 26 e^−^/Å^2^. In fact the Thon ring visibility in the sum of the power spectra from 12 frames of amorphous ice exposure from Fig. 1 is comparable to that seen in the sum of the power spectra of the same number of frames from the amorphous carbon results of Fig. 3 (see of Fig. S.IV in Section C of the Supplementary material). The pattern of the Thon rings from amorphous ice differs from that of carbon, with the amorphous ice signal, as in Fig. 1, strongest around 1/3.7 Å while the corresponding pattern from amorphous carbon is strongest at low spatial frequencies. The 3.7 Å resolution signal in amorphous ice comes from planes of next nearest neighbour oxygen atoms of tetrahedrally coordinated water molecules and the strength of this signal indicates that the ice rules of Bernal and Fowler [11] are applicable in amorphous ice.

The major origin of beam induced motion in an amorphous ice film is from radiolysis rather than knock-on damage [18]. The rate of mass loss from a thin films is observed to be almost independent of film thickness and so most of the radiolysis fragments remain within the film [18]. This so-called cage-effect, allows many of the radiolysis fragments in ice to recombine but some such as O_2_ and H_2_O_2_, will accumulate and re-orient the surrounding water molecules. The successful fits shown in Fig. 5 do however support the simple Gaussian Markov Process description of Section 2.5. In practice irradiation of a plunge frozen amorphous ice film will also result in collective motion of local regions due to stress relief within the film and charging from the emission of secondary electrons.

From our experimental observation we deduce that water molecules, each with a molecular mass of 18 Da, move an RMS distance of ∼1 Å for each e^−^/Å^2^. A typical cryoEM exposure having 25 e^−^/Å^2^ will therefore result in a RMS displacement of water molecules by ∼5 Å. In the same way as the thermal motion of water molecules results in Brownian motion, the beam-induced motion of water molecules will also be expected to displace embedded protein molecules. In a 25 e^−^/Å^2^ exposure a protein molecule of 100 kDa, such as hexokinase, embedded in this film of randomly diffusing molecules would be expected to have an RMS displacement of ∼1.0 Å (hexokinase with a $M_{W}$ of 100 kDa has a 20–40× smaller diffusion coefficient than water, and so should move ∼4–6× less, according to the Stokes-Einstein equation for diffusion [19] in which the diffusion coefficient varies as $1/M_{W}^{1/3}$). For a protein molecule of 25 kDa, the beam-induced random motion would be higher at ∼1.5 Å, and for a ribosome of 2.5 MDa somewhat lower at ∼0.7 Å. This additional random motion produces image blurring that can be represented by an extra B-factor, which for hexokinase would correspond to ∼25 Å^2^, on top of that resulting from other effects such as intrinsic radiation damage to the macromolecular assembly. We can thus conclude that random, Brownian type of beam-induced motion of biological structures is unlikely to be one of the limiting factors in attaining high-resolution structures using single particle cryoEM approaches. Only for very small particles at resolutions of 2 Å or beyond is this type of beam-induced motion likely to be a fundamental limitation.

It can be argued that the observed degree of beam-induced motion of water molecules in amorphous ice during an exposure is actually of positive benefit in cryoEM. If water molecules moved much less during irradiation they would contribute a strong background, just as there is from carbon films, that would decrease the accuracy of the orientation determination in single particle cryoEM. If water molecules moved more then so too would the embedded macromolecules and the images of these would be more blurred.

## Appendix A Supplementary data

Application 1

## Footnotes

Appendix A Supplementary data associated with this paper can be found in the online version at doi:10.1016/j.ultramic.2015.05.017.
