# Supplementary material for: Thon rings from amorphous ice and implications of beam-induced Brownian motion in single particle electron cryo-microscopy
Source: Ultramicroscopy. 2015 Nov;158:26–32. doi: 10.1016/j.ultramic.2015.05.017 (PMC4584428; doi:10.1016/j.ultramic.2015.05.017)
Supplement: Application 1 [file mmc1.pdf]

Supplementary material: Thon rings from amorphous ice and implications of beam-induced Brownian motion in single particle electron cryo-microscopy

G. McMullan, K.R. Vinothkumar, and R. Henderson

MRC Laboratory of Molecular Biology, Francis Crick Avenue, Cambridge, CB2 0QH, U.K.

Contents

A Comparison of $S(u)$ and $W(u)$ from exposure #230804 of Fig.1 **i**

B Statistics of noise whitened power spectra **iv**

C Comparison at 26 e$^-$/Å^2 of Thon rings from amorphous ice with those from pre-irradiated amorphous carbon **v**

D Reduction in Thon ring modulation due to finite sample thickness **vi**

E Calculation of normalised noise power spectrum, $\mathcal{N}(u)$ **xi**

A Comparison of $S(u)$ and $W(u)$ from exposure #230804 of Fig.1

The effect of noise whitening is illustrated in Fig. S.I(a) which compares the spatial frequency dependence of the sum of the circularly averaged power spectrum, $S_{141,1}$ and noise whitened power spectrum, $W_{141,1}$, (using the notation for $W_{\mathrm{M,m}}$ and its equivalent for $S_{\mathrm{M,m}}$ from Eq. (8) obtained from the 141 frames used to generate Fig. 1 of the text. The variation with spatial frequency of $S_{141,1}(w)$ is essentially that of $\mathcal{N}(w)$. The multiplicative factor, Γ, relating $S_{141,1}(w)$ and $\mathcal{N}(w)$ is given in Eq. (7). The measured value for Γ of 684 is within 5% of that from Eq. (7) with a DQE(0) of ∼0.5 and dose of 2.33 e$^-$/pixel. The noise whitening produced by division of $S(w)$ by $\mathcal{N}(w)$ removes this variation, producing a flat background above which the amorphous ice Thon ring signal around 1/3.7Å, now enhanced by $1/\mathcal{N}(w) \sim 24$, can clearly be seen. Noise whitening can be applied to both the sum of the power spectra as shown in

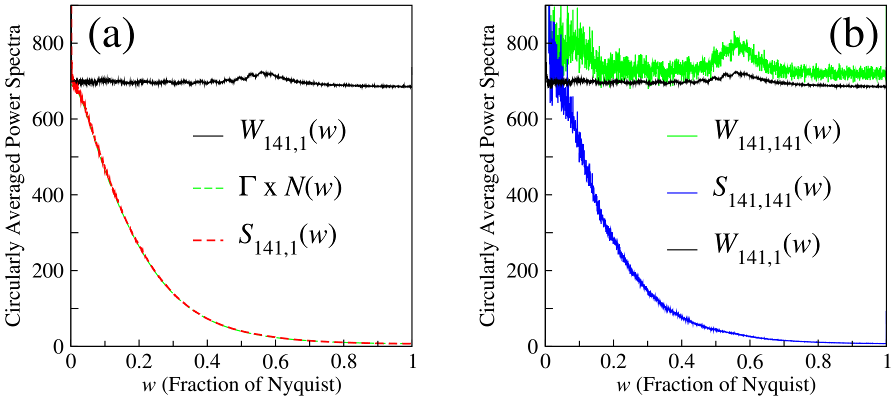

Figure S.I: Comparison of the spatial frequency dependence of the circularly averaged sum of power spectra, $S(w)$, and noise whitened power spectra, $W(w)$, from the 141 frame dose fractionated exposure (image #230804) of amorphous ice used for Fig. 1. The spatial frequency, w is measured as a fraction of the Nyquist frequency (1/2.08Å). In (a), the sum of the power spectra of the individual images ($S_{141,1}(w)$ and $W_{141,1}(w)$) along with $\Gamma\mathcal{N}(w)$ using $\Gamma = 684$ are given. In (b), the circularly averaged power spectrum of the sum of the 141 frames ($S_{141,141}(w)$ and $W_{141,141}(w)$) along with $W_{141,1}(w)$ are shown.

S.I(a) and the power spectrum of the sum of the frames given in S.I(b). The lower noise in the sum of the power spectra provides a more stringent test for the noise whitening procedure; the success of this is clearly illustrated by the featureless flat background in Figs. 1 — 3 of the main text.

Residual correlations in the detector, in particular from the applied linear pixel gain correction, lead to a slight difference in the mean value for the sum of the power spectra versus the power spectrum of the image sum. This is illustrated in Fig. S.I(b) where the value of $W_{141,141}(w)$ is 5% greater than that of $W_{141,1}$.

Fig. S.II(a) compares the spatial frequency dependence of the circularly averaged noise whitened sum of power spectra and that of noise whitened power spectrum of the sum of the frames. For convenience, an offset equal to Nyquist frequency value has been subtracted. The Thon ring signal is bigger in the power spectrum of the sum of the frames but the background noise is also bigger (by a factor of $\sqrt{141} \sim 12$). In Fig. 1 of the main text, the Thon ring signal from the amorphous ice appears stronger in the sum of the power spectra (Fig. 1(b)) rather than in the power spectrum of the sum of the images (Fig. 1(a)). This is because the images in Fig. 1 have been scaled so that background noise has the same amplitude. This effect of this is illustrated in Fig. S.II(b) and is obtained by dividing the circularly averaged power spectrum of the sum frames by $\sqrt{141}$ so that the noise level in the two plots (green and black) is the same.

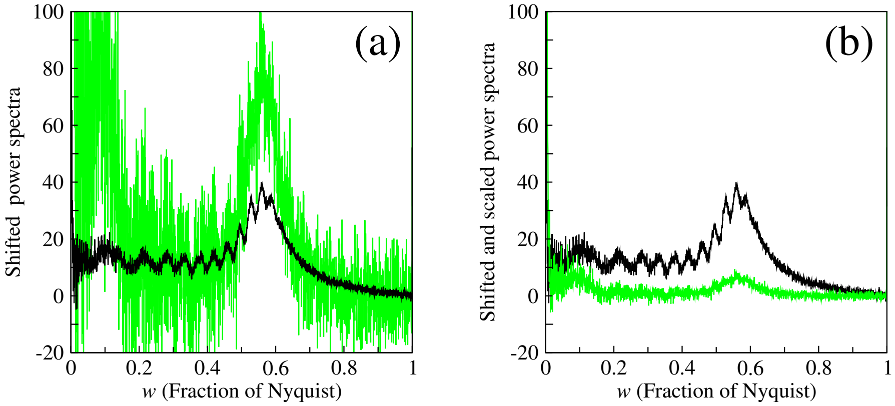

Figure S.II: Comparison of the circularly averaged Thon ring signal from the dose fractionated exposure (image #230804) of amorphous ice as seen in the sum of the noise whitened power spectra (black) with that in the power spectrum of the sum of the image frames (green). The background offset, seen in Fig. S.I(b), has been removed by subtracting the average value near the Nyquist frequency. The results from Fig. S.I(b) shifted in this way are shown in (a) while in (b) the visibility of the Thon ring signal is shown by rescaling the power spectrum of the sum of the images in (a) so that it has the same noise level as sum of the power spectra curve.

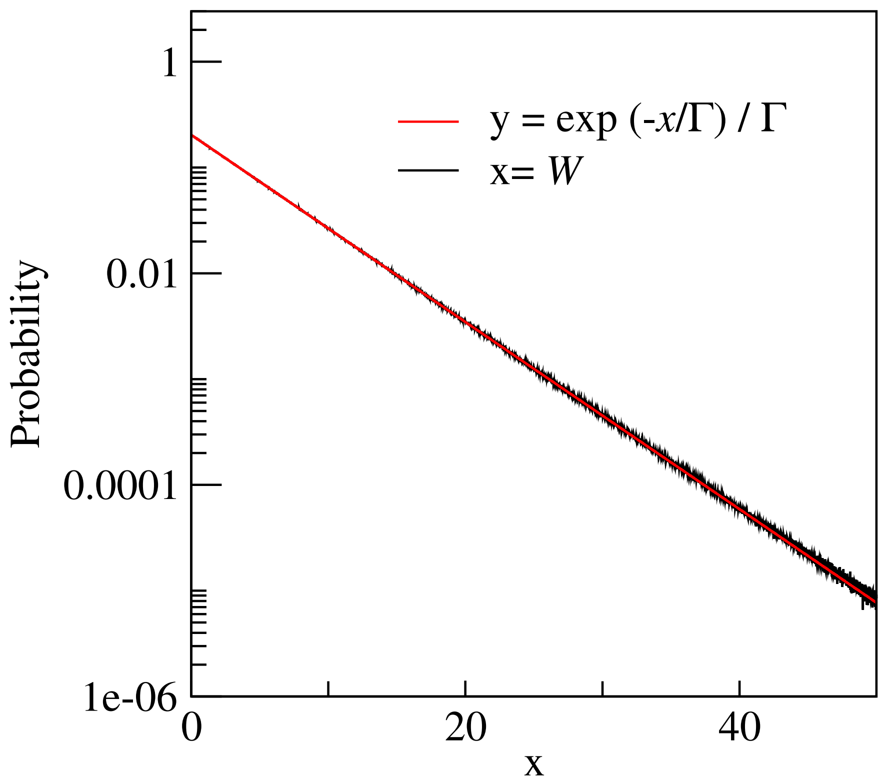

Figure S.III: Histogram of the values in 141 individual frames of noise whitened power spectra from image #230804 of amorphous ice used in Fig. 1. The exponential distribution fit, –, to the measured histogram, —, is with $\Gamma = 4.91$, from Eq. (4).

B Statistics of noise whitened power spectra

As discussed in the text, the distribution of the values in a noise whitened power spectra from the Falcon II is completely described by its mean value $\Gamma \sim d/\mathrm{DQE}(0)$ in which d is the mean number of incident electrons per frame, through the exponential distribution of Eq. (4). To illustrate this, the histogram of values in individual noise whitened power spectra of the 141 frames used in Fig. 1 is given in Fig. S.III. The measured histogram is an excellent fit to an exponential distribution with parameter $\Gamma = 4.91$ (which differs by 5% from the estimate based on $\mathrm{DQE}(0) = 0.5$ and $d = 2.33\,\mathrm{e}^-$/pixel/frame).

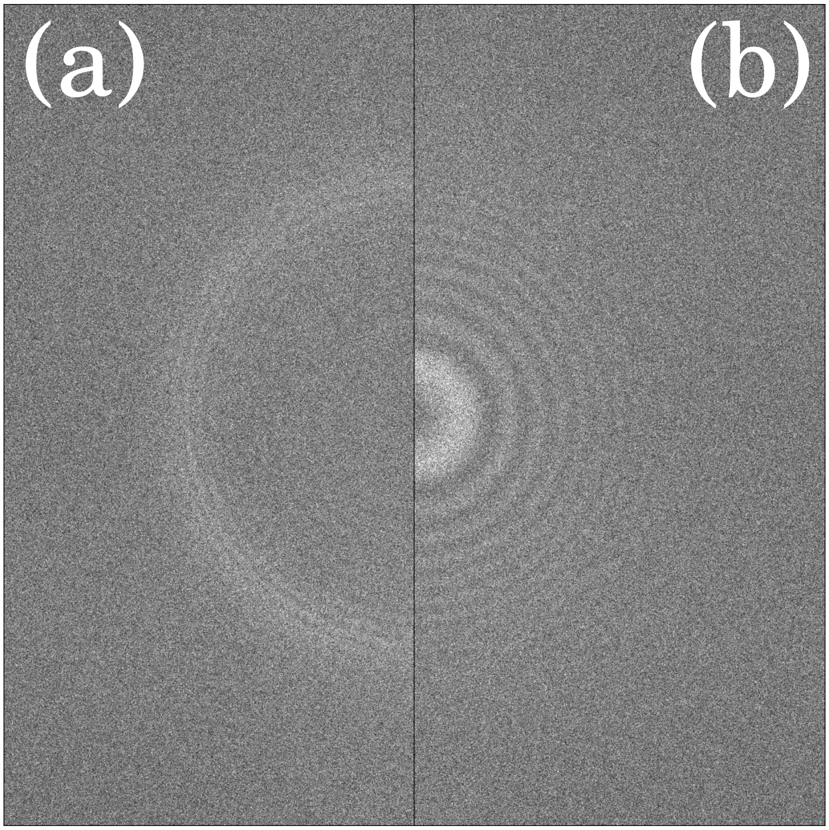

Figure S.IV: (a) Sum of noise whitened power spectra from the first 12 frames of image #230804 from Fig. 1. (b) Sum of noise whitened power spectra from the first 12 frames of the pre-irradiated carbon image used in Fig. 2. The same magnification and total dose ($25.9 \sim 12 \times 2.33/1.04^2\,\mathrm{e^-/Å^2}$) were used in both (a) and (b). The Thon rings from amorphous ice around 1/3.7 Å are stronger than those from the pre-irradiated carbon sample.

C Comparison at $26\,\mathrm{e^-/Å^2}$ of Thon rings from amorphous ice with those from pre-irradiated amorphous carbon

Fig. S.IV(a) shows the Thon rings obtained from the sum of the noise-whitened power spectra from the first 12 frames, corresponding to $25.9\,\mathrm{e^-/Å^2}$ from the 141 used in Fig. 1. For comparison Fig. S.IV(b) shows the corresponding sum of noise whitened power spectra from images of pre-irradiated carbon in Fig. 2. While the Thon rings from carbon are stronger at low spatial frequency, around

1/3.7Å those from the amorphous ice sample are stronger. This is more clearly illustrated in the circular averages shown in Fig. S.V.

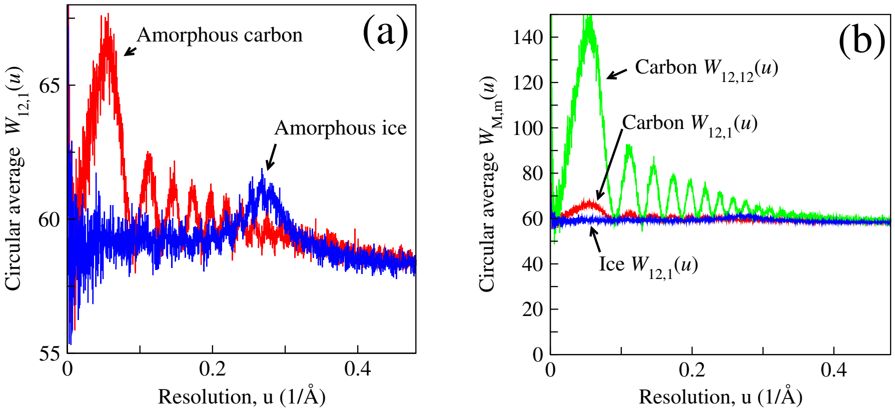

Figure S.V: Comparison of the circular averages of the noise whitened power spectra obtained with $\sim 26\text{e}^-/\text{Å}^2$ from amorphous ice and pre-irradiated carbon. The $W_{12,1}(u)$ circular averages of the amorphous ice (blue) and pre-irradiated carbon (red) sums of noise whitened power spectra are shown in (a). In (b) the corresponding circular average of the power spectrum, $W_{12,12}(u)$, from the sum of the 12 frames of pre-irradiated amorphous carbon is shown.

Fig. S.V(a) shows the circular averages of the sum of the noise whitened power spectra shown in Fig. S.IV. In Fig. S.V(b) the noise whitened power spectrum of the sum of the 12 pre-irradiated carbon frames is compared with corresponding sum of 12 power spectra given in Fig. S.V(a). As the pre-irradiated carbon does not move during an exposure the Thon ring signal adds coherently with increasing number of frames. The Thon ring modulation in the sum of the images, $W_{12,12}(u)$, is 12 times that in the corresponding sum of power spectra, $W_{12,1}(u)$. At the Nyquist frequency the image consists almost entirely of noise and the power spectra of the sum of the images is equal to the sum of the power spectra. The noise in the power spectrum of the sum is however $\sqrt{12}$ times that in the sum of the power spectra.

D Reduction in Thon ring modulation due to finite sample thickness

Biological molecules studied using cryoEM are currently treated as weak phase objects in which the scattered contribution to the exit wavefunction is small and advanced by a phase of $\pi/2$ relative to the transmitted wave [3, 4]. While not strictly valid, this approximation is routinely used to generate ~ 3 Å resolution 3D molecular structures from cryoEM data.

Thon rings result from an additional spatial frequency, u, dependent phase

shift, $\chi(u)$ associated with focusing by the microscope lens of the scattered terms back to the image plane. $\chi(u)$ depends on the image defocus, D, spherical aberration of the lens, C_{s}, and electron wavelength, λ. In the absence of astigmatism it is given by

$$\chi(u) = 2\pi\left(\frac{\lambda}{2}Du^2 - \frac{\lambda^3}{4}C_{\mathrm{s}}u^4\right). \tag{S.1}$$

In the image plane the electron wavefunction, $\Psi(\boldsymbol{x})$, is

$$\Psi(\boldsymbol{x}) \sim 1 + i\int \mathrm{e}^{i(2\pi\boldsymbol{x}.\boldsymbol{u}+\chi(u))}V_p(\boldsymbol{u})\,\mathrm{d}\boldsymbol{u} \tag{S.2}$$

where $V_p(\boldsymbol{u})$ is proportional to the Fourier transform of the projected potential of the atoms in the sample. The probability of an electron being detected at a given point, $\boldsymbol{x}$, is given by $|\Psi(\boldsymbol{x})|^2$ and to first order in the scattered terms is

$$|\Psi(\boldsymbol{x})|^2 \sim 1 - 2\int \mathrm{e}^{i2\pi\boldsymbol{x}.\boldsymbol{u}}V_p(\boldsymbol{u})\sin\chi(u)\,\mathrm{d}\boldsymbol{u}. \tag{S.3}$$

A pixel value, f_{rs}, is given by the integral over the (r,s)-pixel of Eq. (S.3) convoluted with the detector response function. The Fourier components given by Eq. (1) are proportional to $\sin\chi(u)$ resulting in the $\sin^2\chi(u)$ Thon ring modulation term seen in power spectra such as shown in Figs. 1-3.

The validity of using the projected potential breaks down [1] as the thickness of a sample increases. Thicker samples can however be thought of as consisting of slices at different z heights perpendicular to the beam. The projected potential of each slice is used but they are now multiplied by $\chi(u)$ with defocus $D+z$ which is convenient to write as $\chi(u, D+z)$. The spatial frequency at which the zeros in $\chi(u, D+z)$ occur depends on z and summing over different z heights results in the washing out of the Thon ring modulation at higher spatial frequencies[1]. In the limit of infinitesimally thin slices the Fourier component, $F_t(u)$ of a sample with thickness t and average defocus of D is given by the integral

$$F(u) = \int_{-t/2}^{+t/2} V_p(\boldsymbol{u}, z)\sin\chi(u, D+z)\,\mathrm{d}z \tag{S.4}$$

in which $V_p(u,z)$ is the projected potential density in a slice offset from the mean by a distance z. The power spectrum $S(u)$ at u is proportional to $|F(u)|^2$ and given by

$$S(u) \propto \int_{-t/2}^{+t/2} V_p^*(\boldsymbol{u}, z)\sin\chi(u, D+z)\,\mathrm{d}z \times \int_{-t/2}^{+t/2} V_p^*(\boldsymbol{u},' z)\sin\chi(u, D+z')\,\mathrm{d}z' \tag{S.5}$$

$$\propto <|V_p(\boldsymbol{u})|^2> \int_{-t/2}^{+t/2} \sin^2\chi(u, D+z)\,\mathrm{d}z \tag{S.6}$$

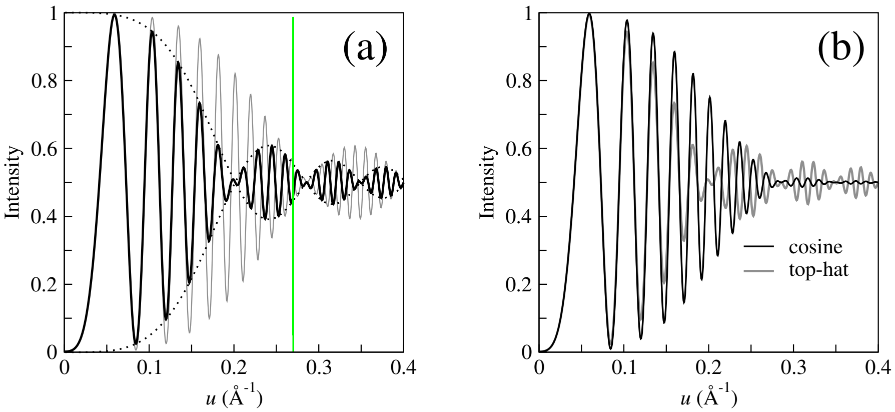

Figure S.VI: (a) Plot showing the effect of finite thickness on the Thon ring modulation for 300 keV electrons with a defocus of 7070 Å, $C_s = 2.7\,$mm and ice thickness of 1250 (black) and 630 (grey), respectively. The vertical green line indicates a resolution of 1/3.7 Å while the black dotted lines indicate the $\sin(\xi)/\xi$ modulation envelope. (b) Plot show the effect of replacing the top-hat profile (grey) of the 1250 Å thick sample in (a) with a cosine profile (black). The use of a cosine profile results in a more gental damping of the Thon ring modulation and supresses ringing at higher spatial frequencies.

with the double integral reducing to a single integral as the layers are assumed to scatter independently while $<|V_p(\boldsymbol{u})|^2>$ is independent of z since the layers are assumed to be equivalent. Carrying out the integral gives

$$S(u) \propto <|V_p(\boldsymbol{u})|^2> \frac{t}{2}\Big[1 - \mathrm{sinc}(\xi)\cos(2\chi(u, D))\Big] \tag{S.7}$$

in which $\mathrm{sinc}(\xi) = \sin(\xi)/\xi$ and $\xi = \pi\lambda u^2 t$. The strength of the power spectrum is proportional to the sample thickness and the $\mathrm{sinc}(\xi)$ term damps out the Thon ring modulation at a given spatial frequency, u, as the thickness of the sample increases. In very thick samples the Thon ring modulation will disappear but the average value will remain. In determining the movement of water molecules in ice the behaviour of either the Thon ring modulation or the absolute contribution to the power spectrum from the ice scattering can be used. The latter was used to obtain the results shown in Fig. 5 of the main text.

In the discussion in the main text the ice thickness for Fig. 1 was estimated to between 1000 and 1500 Å. The effect of the variation in defocus through a sample of this thickness described by Eq. (S.7) is illustrated in Fig. S.VI(a) which shows the behaviour as a function of spatial resolution (assuming $<|V_p(\boldsymbol{u})|^2>$ is a constant) with an average defocus of 7070 Å using 300 keV electrons ($\lambda = 0.0197$Å) in samples of 1250 Å (black) and 630 Å (grey). The vertical green line indicates a resolution of 3.7 Å and at this resolution $|\sin(\xi)/\xi| = 0.1$ for both thicknesses. However for 1250 Å this resolution is beyond the first zero

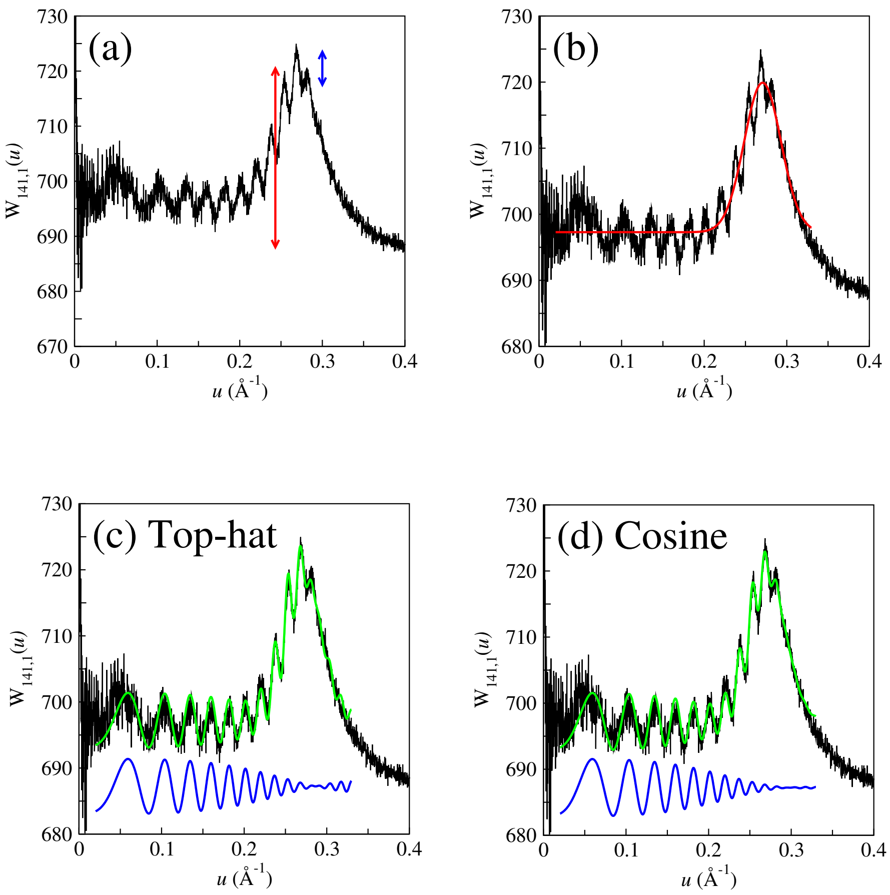

Figure S.VII: (a) Measured circularly averaged Thon ring modulation from Fig. 1. The red line indicates an average value above the background of 33 while the blue line indicates a peak to peak value of 6 corresponding to a 9% modulation. (b) Background fit (red) to the circularly averaged Thon ring signal using Eq. (S.10) consisting of a constant and a single Gaussian peak centred around 0.27 Å^{-1}. (c) Shows a fit to the based on a top-hat profile through the sample using Eq. (S.7). The fit shown in green uses a thickness of 619 Å and a defocus of 7061 Å. The contribution without the amplification from the Gaussian peak at 0.27 Å^{-1} is shown offset in blue. (d) Shows a fit based on a cosine profile through the sample using Eq. (S.9) with thickness 1153 Å and defocus of 7060 Å. The corresponding contribution without the amplification from the Gaussian peak at 0.27 Å^{-1} is shown offset in blue.

of $\sin(\xi)/\xi$. The measured Thon ring modulation around 1/3.7 Å is $\sim 9\,\%$ (see Fig. S.VII) but contains no evidence for a node in the Thon ring modulation at lower resolution spatial frequency.

The $\sin(\xi)/\xi$ ringing arises from the fixed thickness and uniform (top-hat) profile of the scattering in Eq. (S.7). In amorphous ice, the dynamic disorder resulting from the escape of radiolysis by-products will reduce the contribution from boundaries and a shaped profile reflecting this may be more appropriate. In particular adding a cosine profile, i.e.,

$$w(z) = 1 + \cos(2\pi z/t) \qquad \text{for} \qquad z \in [-t/2, t/2] \tag{S.8}$$

to the integral in Eq. (S.6) gives

$$S(u) \propto <|V_p(\boldsymbol{u})|^2> \frac{t}{2}\left[1 - \mathrm{sinc}(\xi)\,\frac{\pi^2}{\pi^2 - \xi^2}\cos(2\chi(u, D))\right] \tag{S.9}$$

where as in Eq. (S.7) $\xi = \pi\lambda u^2 t$. As shown in Fig. S.VI(b) the result of using a cosine rather than a top-hat profile is to reduce the initial damping and almost completely remove oscillations in the Thon ring modulation envelope at higher spatial frequencies.

Fig. S.VII shows the circular average of Thon ring modulation from Fig. 1. The experimental estimation of the Thon ring modulation is illustrated in Fig. S.VII(a). The increased scattering from amorphous ice around 1/3.7 Å leads to a peak in, and corresponding amplification of, the Thon ring signal.

The absence of long range order in amorphous ice makes it possible to model the scattering term, $<|V_p(u)|^2>$, in Eq. S.7 and Eq. S.9 using a constant background with a Gaussian peak centred around 1/3.7 Å, i.e.,

$$<|V_p(u)|^2> \approx a_0(1 + a_1 \exp(-(u - a_2)^2/2a_3^2)). \tag{S.10}$$

The resulting four parameter, $a_0, \ldots a_3$, fit to $W_{141,1}(u)$ is shown in Fig. S.VII(b).

The difference between measured circularly averaged $W_{141,1}(u)$ and the fit shown in Fig. S.VII(b) can be used to find an initial estimate for the defocus, D. Using these as initial values the thickness, t, can then be estimated using fits with either the top-hat (Eq. (S.7)) or cosine (Eq. (S.9)) approximations. A fit using the top-hat profile of Eq. (S.7) results in an estimate for the ice thickness of 619 Å. The result shown in Fig. S.VII(c) is a good fit to the experimental data though the damping at higher frequencies is too small. The latter could be just due to not including effects of partial coherence or beam divergence. A fit using the cosine profile of Eq. (S.9) results in an estimate of 1153 Å for the ice thickness and gives very little modulation at higher frequencies. Again a good fit to the measured result is obtained. In both cases the amplification from the peak in $<|V_p(u)|^2>$ is important for describing the measured behaviour.

E Calculation of normalised noise power spectrum, $\mathcal{N}(u)$

The energy lost by incident high energy electrons passing through the lightly doped semi-conducting sensitive layer of a CMOS sensor such as the Falcon II, produces electron/hole pair excitations. The sensitive layer is surround by heavily P-doped layers into which the holes are free to escape but the negatively charged electron excitations are trapped due to the potential resulting from the differential doping. An incident high energy electron is recorded via the voltage drop on reverse biased diodes formed by a heavily N-doped implants on the surface of the sensitive layer resulting for collection of the electron excitations. The number of electron-hole pairs generated by an incident high energy electron, and hence the signal, depends on the length of the incident electrons trajectory through the sensitive layer. The thick sensitive layer of the Falcon II results in a large average signal but the thickness of the sensitive layer allows electrons to diffuse relatively large distances before they are finally collected. This diffusion results in a poor modulation transfer function, MTF, even with the relatively large ($14\,\mu$m) pixel size of the Falcon II. The diffusion acts as a low-pass filter and while it lowers the MTF it has only a small effect on the detective quantum efficiency, DQE. In other words the spatial distribution of the signal deposited in the detector by an incident electron, described by the point spread function (PSF), is mainly determined by electron/hole diffusion and not scattering of the primary incident electron (at least for incident 300 keV electrons). The Fourier transform of the point spread function determines the intrinsic spatial frequency response of the detector and its square describes the noise power spectrum. In a real pixellated detector the noise power spectrum also includes pixel modulation terms of the form $\sin(x)/x$ from integration over the pixel and contributions aliased back from spatial frequencies beyond the Nyquist frequency of the pixellated sampling.

As shown in [2] the $\mathcal{N}(u)$ for the Falcon II detector is to a good approximation given by

$$\mathcal{N}(u,v) = \sum_{\mathbf{G}} \left(\sin(\pi a u_G) \sin(\pi a v_G)\, \mathrm{MTF}_0(\sqrt{u_G^2 + v_G^2}) \right)^2 \tag{S.11}$$

where a is the pixel pitch, the sum is over the low order reciprocal lattice vectors needed to describe the aliasing, u_G and v_G are the spatial frequency components i.e., $u_G = u + G_u$, and MTF_0 is the Fourier transform of the intrinsic PSF. In [2] $\mathcal{N}(u,v)$ was calculated from the MTF_0 obtained from the experimentally measured MTF. While this works well (reducing the variation in the power spectra to a few percent), the higher signal to noise in the longer exposures used in the present work show small systematic variations. The results presented in Figs. 1-3 were therefore obtained using a fit to a radial function $\phi(u)$, effectively $\mathrm{MTF}_0(u)^2$, from zero to twice the Nyquist frequency of the measured noise power spectra using the functional form in Eq. (S.11).

The simplest way to calculate $\mathcal{N}(u,v)$ is to average the noise power spectra of many frames and apply the symmetry from the square pixel array. This leaves some residual noise and the problem of finding the overall normalisation. In the present work a radial function, $\phi(u)$, was used to fit the symmetrized sum of the noise power spectra via

$$S(u,v) \sim \sum_{\mathbf{G}} \sin^2(\pi a u_G) \sin^2(\pi a v_G)\, \phi(\sqrt{u_G^2 + v_G^2}). \tag{S.12}$$

The overall normalisation was found by using a single Gaussian function in $\phi(u)$ to fit the low spatial frequency limit and a smooth general fit to the remainder via a b-spline fit that was penalty weighted to ensure smoothness and non-negative values. A more detailed description of this and its implementation will be presented elsewhere.

References

[1] David J. DeRosier. Correction of high-resolution data for curvature of the ewald sphere. *Ultramicroscopy*, 81(2):83 – 98, 2000.

[2] G. McMullan, A.R. Faruqi, D. Clare, and R. Henderson. Comparison of optimal performance at 300keV of three direct electron detectors for use in low dose electron microscopy. *Ultramicroscopy*, 147(0):156–163, 2014.

[3] Ludwig Reimer and Kohl Helmut. *Transmission Electron Microscopy - Physics of Image Formation.* Springer-Verlag New York, fifth edition, 2008.

[4] John C. H. Spence. *High-Resolution Electron Microscopy.* Oxford University Press, Oxford, fourth edition, 2013.
